# The Strategies to Support the COVID-19 Vaccination with Evidence-Based Communication and Tackling Misinformation

**DOI:** 10.3390/vaccines9020109

**Published:** 2021-02-01

**Authors:** Piotr Rzymski, Leszek Borkowski, Marcin Drąg, Robert Flisiak, Jacek Jemielity, Jacek Krajewski, Agnieszka Mastalerz-Migas, Andrzej Matyja, Krzysztof Pyrć, Krzysztof Simon, Michał Sutkowski, Jacek Wysocki, Joanna Zajkowska, Andrzej Fal

**Affiliations:** 1 Department of Environmental Medicine, Poznan University of Medical Sciences, 60-806 Poznań, Poland; 2 Integrated Science Association (ISA), Universal Scientific Education and Research Network (USERN), 60-806 Poznań, Poland; 3 The Foundation “Together in Disease”, 05-252 Dąbrówka, Poland; c@data.pl; 4 Department of Chemical Biology and Bioimaging, Wroclaw University of Science and Technology, 50-370 Wroclaw, Poland; marcin.drag@pwr.edu.pl; 5 Department of Infectious Diseases and Hepatology, Medical University of Bialystok, 15-540 Białystok, Poland; robert.flisiak1@gmail.com; 6 Centre of New Technologies, University of Warsaw, 02-097 Warsaw, Poland; j.jemielity@cent.uw.edu.pl; 7 The Federation of Healthcare Employers’ Unions “Porozumienie Zielonogórskie”, 65-048 Zielona Góra, Poland; jkrajewski52@gmail.com; 8 Department of Family Medicine, Wroclaw Medical University, 50-367 Wroclaw, Poland; agnieszka.mastalerz-migas@umed.wroc.pl; 9 Supreme Medical Council of the Polish Supreme Chamber of Physicians and Dentists, 00-764 Warsaw, Poland; andrzej.matyja@hipokrates.org; 10 2nd Department of General Surgery, Jagiellonian University Medical College of Krakow, 30-688 Kraków, Poland; 11 Virogenetics Laboratory of Virology, Malopolska Centre of Biotechnology, Jagiellonian University, 30-387 Krakow, Poland; k.a.pyrc@gmail.com; 12 Department of Infectious Diseases and Hepatology, Wrocław Medical University, 51-149 Wrocław, Poland; krzysztof.simon@umed.wroc.pl; 13 College of Family Physicians in Poland, 00-209 Warsaw, Poland; doktormjs@gmail.com; 14 Faculty of Medicine, Lazarski University, 02-662 Warsaw, Poland; 15 Department of Preventive Medicine, Poznań University of Medical Sciences, 60-179 Poznań, Poland; jawysocki@pro.onet.pl; 16 Department of Infectious Diseases and Neuroinfections, Medical University of Bialystok, 15-540 Białystok, Poland; zajkowsk@umb.edu.pl; 17 Collegium Medicum, Warsaw Faculty of Medicine, Cardinal Stefan Wyszyński University, 01-938 Warsaw, Poland

**Keywords:** COVID-19, vaccine, science communication, vaccine hesitancy, misinformation

## Abstract

COVID-19 vaccinations are about to begin in various countries or are already ongoing. This is an unprecedented operation that is also met with a loud response from anti-vaccine communities—currently using all available channels to manipulate public opinion. At the same time, the strategy to educate on vaccinations, explain their mechanism of action, and build trust in science is subdued in different world parts. Such actions should go much beyond campaigns promoting the COVID-19 vaccines solely on the information provided by the health institutions and national authorities. In this paper, actions provided by independent expert groups needed to counteract the anti-vaccine propaganda and provide scientific-based information to the general public are offered. These actions encompass organizing groups continuously communicating science on COVID-19 vaccines to the general public; tracking and tackling emerging and circulating fake news; and equipping celebrities and politicians with scientific information to ensure the quality of messages they communicate, as well as public letters, and statements of support for vaccination by healthcare workers, recognized scientists, VIPs, and scientific societies; and no tolerance to false and manipulated claims on vaccination spread via traditional and social media as well as by health professionals, scientists, and academics. These activities should be promptly implemented worldwide, regardless of the current status and availability of the COVID-19 vaccine in a particular region. If we are about to control the pandemic for the sake of public benefit, it is high time to collectively speak out as academic and medical societies with support from decision-makers. Otherwise, the battle will be lost to those who stand against scientific evidence while offering no feasible solution to the problem.

## 1. Introduction

The novel coronavirus disease (COVID-19), caused by severe acute respiratory syndrome coronavirus 2 (SARS-CoV-2) and reported for the first time in late December 2019, was declared by the World Health Organization as a pandemic in March 2020 [1,2]. The uncontrolled spread of the disease has overwhelmed the healthcare system, caused a global economic crisis, led to disturbances in education, induced public fears and anxiety, forced at least 4 billion people into isolation in nationwide lockdowns in different world parts, magnified pre-existing psychological issues, and affected life on every possible level [3,4,5,6,7,8,9]. By the end of 2020, over 80 million cases and over 1.76 million deaths due to COVID-19 were reported.

The pandemic has also been met with an unprecedented and rapid response from the scientific community with a massive publication output [10,11,12], and a number of the repurposed pharmaceuticals such as remdesivir, arbidol, chloroquine, darunavir, dexamethasone, favipiravir, hydroxychloroquine, lopinavir, interferons, ribavirin, ritonavir, and tocilizumab have been extensively tested [13,14,15]. There has also been a substantial effort to seek novel pharmaceutical agents [16,17] and the use of convalescent plasma [18,19]. Despite all of these efforts, no universal treatment method for COVID-19 was available by the end of 2020. There is also a scientific consensus that naturally acquired herd immunity is not a feasible strategy for pandemic management and would be a “dangerous fallacy unsupported by scientific evidence” [20,21,22].

At the same time, a great effort has been put forward to develop vaccine candidates using different approaches encompassing traditional live attenuated and inactivated vaccines, and modern solutions employing viral vectors, mRNA, DNA, single proteins, and virus-like particles as carriers [23,24]. The first candidate to enter the phase I clinical trial was mRNA-1273, developed by U.S.-based Moderna Therapeutics and administrated to volunteers as early as 16 March 2020 [24]. By the end of November 2020, interim results from phase III clinical trials were already announced [25] and, in December 2020, the BNT162b2 mRNA vaccine (brand name Comirnaty), developed by BioNTech and manufactured by Pfizer, was approved for use in the United Kingdom, the Kingdom of Bahrain, Canada, Saudi Arabia, the European Union, and the USA [26,27]. Other candidates, such as mRNA-1273 (COVID-19 Vaccine Moderna) and ChAdOx1 (AZD1222), were also approaching assessment and potential approval in different geographical regions [28]. The decision to initiate COVID-19 vaccination programs gives hope that the pandemic can be brought under control and is met with relief from a number of healthcare workers, who have been overworked for the last couple of months [29]. However, some studies reported high vaccine hesitancy among health professionals or some of their occupational categories in selected world regions [30,31,32].

**Figure 1:**
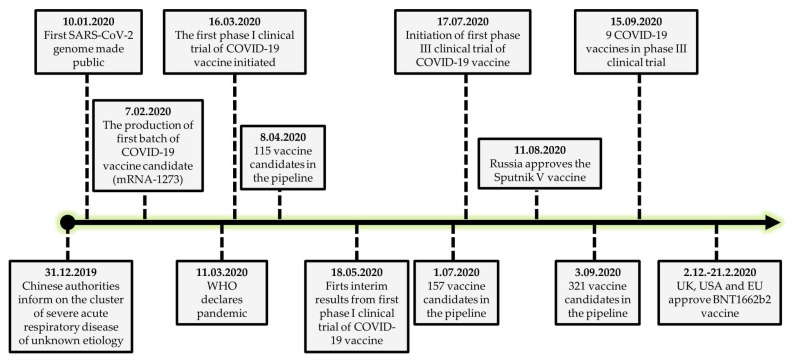
The general timeline of COVID-19 vaccine development by the end of 2020.

This unprecedented speed at which the COVID-19 vaccine was made available (Figure 1) is thanks to years of research, technological advances and platforms enabling a faster manner of vaccine development, significant funding that allowed to run multiple trials in parallel, and regulatory institutions working at a higher pace when considering the applications to initiate specific testing phases and evaluating their results [33]. However, it must be stressed that the COVID-19 vaccine may not be available in some geographical regions by 2022 [34].

With COVID-19 vaccines made now available for use in some regions, the fight against COVID-19 is now entering another phase. To stop the pandemic spread, a large proportion of the population, estimated between 60% and 90% depending on several factors, e.g., vaccines efficacy and the reproduction number [20,35,36], needs to acquire immunity. There is a great need for effective communication on COVID-19 vaccination that goes beyond a usual effort to reach this goal. Without it, vaccine hesitancy will take over, further magnified by the actions of those who ideologically oppose the vaccination strategies. It is particularly important for the scientific community to present consistent, high-quality information accessible for non-specialists to tackle circulating fake news.

This paper presents the main strategies, beyond those put forward by health authorities, required to reach the general public and support it in the decision-making process regarding the COVID-19 vaccination. These actions should be promptly implemented worldwide with the help of independent experts, regardless of the current status of the COVID-19 vaccine (vaccine already in use or to be approved and made available) in any particular world region.

## 2. Strategies to Provide Evidence-Based Information on COVID-19 Vaccines

**Figure 2:**
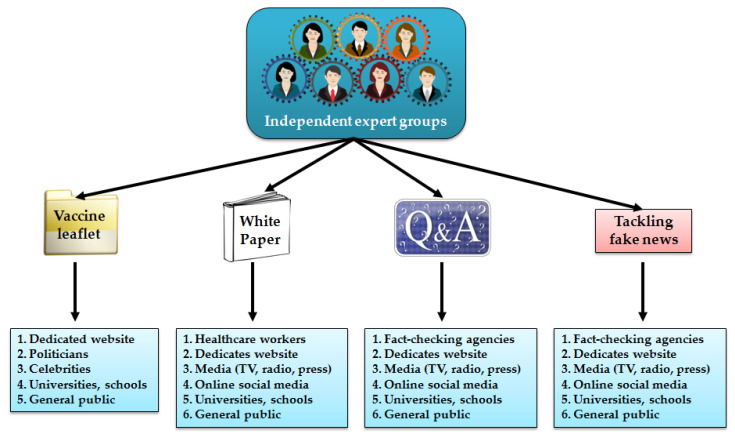
The main strategies to increase the COVID-19 vaccine awareness are realized by independent expert groups and the main recipients of offered actions.

With the COVID-19 vaccination made non-mandatory in different parts of the world, high levels of public agreement and understanding will be required to make these actions successful. While most of the world still waits to use the vaccines outside of clinical trials, anti-vaccine movements are already using every opportunity to generate hesitancy and instill a lack of confidence in science [37]. This comes with widespread access to the internet and online social media forums that facilitate the uncontrolled spread of fake news. Simultaneously, the vaccines based on modern technologies, such as messenger RNA platform or recombinant viral vectors, can be easily targeted to trigger public fear, especially considering that there has already been a flood of scientifically unsupported claims about COVID-19 casting doubts on health-protective-behaviors during a pandemic [38,39]. There is no doubt that the anti-vaccine movements will now take every chance to manipulate public opinion. This already happens with false claims that the vaccine administration modifies the human genome, induces irreversible damage to human health, contains human immunodeficiency virus particles, or implants tracking chips. Notably, the baseline willingness for vaccination is not high enough in many countries [40,41] and will not improve without effective and pro-active campaigns that also keep a constant watch to counteract misinformation. The general public must be given access to the pivotal information on the authorized vaccines, and that their approval is based on the evidenced benefits that outweigh the potential risks of vaccine administration [42]. Moreover, while some world regions are expected to wait longer for the broad availability of vaccines [34], the spread of misinformation and fake news may have disastrous effects on the decision-making process. This may be particularly true for less developed regions, e.g., Africa, where science communication regarding COVID-19 has already been subdued, while strong cultural and religious beliefs, as well as limited access to education, support a spread of myths and misconceptions that exist among the public and even political leaders [39]. Therefore, it is high time to act now, regardless of the status of the vaccine in a particular country and its social and economic development level (Figure 2).

### 2.1. Organizing Expert Groups Communicating Science on COVID-19 Vaccines

It is highly insufficient to base campaigns promoting the COVID-19 vaccines solely on the information provided by health and national authorities. Additional expert activities need to come forward and employ online social and traditional media to provide accurate, yet understandable information for non-specialists that covers the mechanism of vaccines’ action, the research process, approval regulations, and individual and public benefits of vaccinations against COVID-19 as well as their safety profile.

To ensure visibility and high quality, all such initiatives need to be supported by organized groups of high-rank, independent experts without any conflicts of interest and with professional support from experienced public relations agencies and local media. Partnering with public institutions, such as universities, libraries, and schools, is also highly recommended as they constitute a valuable source through which evidence-based information can be communicated to the general public [43,44]. It is highly advised that these expert groups cover a wide range of scientific areas, including infectious diseases, epidemiology, virology, biochemistry, vaccinology, medical biology, as well as public health. Importantly, physicians of primary care should also constitute a part of such groups as they have direct contact with the largest number of patients and have the highest share in health services provided by the entire healthcare system. They also constitute the common source of information for medical personnel employed in thousands of clinics across the country.

The first aim of an expert group should be to publish the White Paper that informs one on the main features of COVID-19 and SARS-CoV-2, the related epidemiology, types of vaccines, their mechanism of action, testing, approval procedures, logistic issues, and risks and benefits of vaccination. Such a White Paper should be strictly based on the newest existing scientific data, but presented in plain language, preferentially accompanied by explanatory graphics. It is pivotal to make the White Book available to all healthcare workers in the country as they are critical to support the decision-making of their patients. Moreover, as healthcare workers are a priority group for COVID-19 vaccination, they are also role models for the general public. The White Paper should also be made available online to all national media as well as to general public to provide them with accurate, evidence-based information to decrease the risk of fake news on vaccines being spread. One of the first examples of such action was the publication of the Polish White Paper on COVID-19 vaccines prepared by an expert group of health professionals and scientists under the initiative “Science against Pandemic” [45].

To increase its impact, the White Paper can be accompanied by infographics, explanatory videos, and Q&A sessions made available via online websites and social media. Importantly, it should be updated as soon as updated evidence on vaccines emerges or new candidates are authorized for use. Such updates should always be communicated through partners and the media to the general public.

If the White Paper is prepared in a non-native English-speaking country, an expert group should consider translating it into English language and making it publicly available. This will help to reach out to countries with poorer resources and provide the local experts with ready-to-use information. The local authorities and health institutions in such countries should be directly informed about such a publication to gain their attention.

### 2.2. Tracking and Tackling Fake News on COVID-19 Vaccines

The COVID-19 pandemic has been accompanied by an enormous and prevalent spread of fake news, misinformation, and conspiracy theories—a phenomenon for which the World Health Organization coined the term ‘infodemic’ [38,46,47]. It has been undermining trust in health institutions, programs, and expert opinions, resulting in lower adherence to pivotal sanitary recommendations and contributing to the SARS-CoV-2 transmission and deaths [48]. With the emergence of COVID-19 vaccines, the spread of fake news and deliberate manipulations still increased. Therefore, it is recommended that the above-mentioned expert groups, in collaboration with a professional public relations agency, will continuously identify and even predict possible fake news concerning COVID-19 vaccines and address them as soon as possible via all possible channels, including public as well as social media. Personal engagement of high-ranked scientists is crucial as the outreach of professional journalists is limited by the worldview of the part of the audience to which they usually turn. It is also recommended to extensively comment on all fake news on the COVID-19 vaccines in a separate paper in which they are explained and counteracted with the scientific evidence, but in plain language, in a manner understandable to non-specialists. The publication should be made available online and should also be sent to all national media as well as continuously updated each time novel false claims emerge. It is also pivotal for an expert group to co-operate with fact-checking agencies and local media and provide them with information tackling the circulating fake news. In order to avoid highly emotional reactions to the fact-checking materials that can subsequently generate distrust [49], it is advised to avoid personal attacks on individuals spreading the false claims, provide commentaries from a diverse group of experts, and support the information with high-quality scientific references. The emphasis should be put on providing facts and explanations, not undermining the credibility of others in a direct manner.

### 2.3. Equipping Celebrities and Politicians with Scientific Information on COVID-19 Vaccines

The immense influence of celebrities (e.g., recognized singers, actors, television show hosts, and professional athletes) on decisions made by the general public is well established, thus they are frequently employed to shape consumers’ views [50,51]. At the time of COVID-19, it is reasonable to convince and support celebrities to promote pro-vaccine campaigns and vaccination programs. This may include VIPs’ official statements via online social or national media, as well as taking their vaccine in public. It is pivotal to recruit such individuals from different age groups to reach the public in the broadest manner. Convincing politicians representing different sides of the political scene to support vaccination is absolutely necessary to avoid vaccination becoming a politically sensitive issue. However, extreme care must be taken to ensure the highest quality of the message conveyed by these individuals. The mentioned expert group needs to supervise the merits and provide VIPs with sufficient vaccine information understandable to the non-specialist.

### 2.4. Supporting the COVID-19 Vaccination through Public Letters and Statements

Significant decisions should be made under the influence of expert advice [52,53]. It is beyond any doubt that this is also true for the take-or-not-to-take decision on the COVID-19 vaccine. All vaccinated scientists, medical practitioners, and VIPs can be encouraged to share the background of their decision and all vaccine-related experiences. However, an even broader effect can be achieved by the open letters of support for vaccination signed by a relevant number of healthcare workers, recognized scientists, VIPs, and scientific societies. Such actions will positively affect attitudes towards COVID-19 vaccines in the general public. Various statements have already been made during the COVID-19 pandemic by the scientific communities and have been readily used by media outlets positively shaping public opinion [12,22,54,55,56]. This is the right time for communities of biomedical researchers and clinical practitioners to step forward and support the scientifically-driven action on the pandemic through the vaccination programs.

### 2.5. No Tolerance to False and Manipulated Claims on COVID-19 Vaccines

False claims about COVID-19 vaccines made by health professionals, scientists, and academics should no longer be tolerated. They must be considered as unethical and treated as such on a legal level by medical councils, universities, and scientific institutes. Democracy does allow for free speech, but it does not exempt those who spread false claims from its consequences. Those who run online social media should also take responsibility, ensure that content on the COVID-19 vaccines is accurate, and prevent algorithms from amplifying scientifically unsupported claims [57,58]. This can be achieved, inter alia, by adding a tool allowing the users to specifically report the content suspected to spread fake news on COVID-19 and vaccines. The social media operators should then consult with a group of experts to verify the accuracy of the reported content. It is also pivotal to ensure that traditional media communicate information and news on vaccinations in a responsible manner. The COVID-19 pandemic has already seen a positive role of mass media in reinforcing public health communication and recommendations on hygiene practices [59]. However, there are examples, particularly in the early stages of a pandemic, of how media coverage could exaggerate social anxiety by using fearful references such as ‘killer virus’; ‘deadly virus’; ‘alarming spread’; or ‘highly contagious, deadly disease’ [60,61,62]. An effort should be made to avoid hysterical headlines and click-bait techniques to generate higher revenue. Potentially sensitive material should be accompanied by an expert commentary to minimize the risk of misinterpretation. For example, cases of severe adverse reactions to vaccinations (e.g., anaphylaxis and other severe allergic reactions) should always be put in the context of the general frequency of such events in the population and potential background causes to lower a risk of false impressions, increasing social fears over the vaccination and fuelling anti-vaccine movements.

## 3. Conclusions

Taken together, in our opinion, the typical communication limited to national authorities and health institutions will not be sufficient to successfully reach the general public and decrease vaccine hesitancy, because COVID-19 by itself is not a typical situation and the pandemic has already seen a massive flood of misinformation and fake news. We postulate an increasing need for organized actions conducted in a professional manner by recognized scientists, but directed to non-specialists and understandable to the general public. These actions are pivotal regardless of the status of the COVID-19 vaccine in a particular country, although they should be especially intensified before and in the early phases of the vaccination programs. Widespread vaccination is the most effective, if not the only, way to control the COVID-19 pandemic. It thus requires high acceptance of the general public. For the sake of the entire society’s benefit, it is high time to collectively speak out as academic and medical societies to support decision-makers. Otherwise, the battle can be lost to those who stand against scientific evidence, endangering public health while not offering any feasible solution to the problems posed by SARS-CoV-2.
